# PredLnc-GFStack: A Global Sequence Feature Based on a Stacked Ensemble Learning Method for Predicting lncRNAs from Transcripts

**DOI:** 10.3390/genes10090672

**Published:** 2019-09-03

**Authors:** Shuai Liu, Xiaohan Zhao, Guangyan Zhang, Weiyang Li, Feng Liu, Shichao Liu, Wen Zhang

**Affiliations:** 1 College of Informatics, Huazhong Agricultural University, Wuhan 430070, China; 2 School of Computer Science, Wuhan University, Wuhan 430072, China

**Keywords:** lncRNA prediction, genetic algorithm, stacked ensemble learning, global sequence features, feature selection

## Abstract

Long non-coding RNAs (lncRNAs) are a class of RNAs with the length exceeding 200 base pairs (bps), which do not encode proteins, nevertheless, lncRNAs have many vital biological functions. A large number of novel transcripts were discovered as a result of the development of high-throughput sequencing technology. Under this circumstance, computational methods for lncRNA prediction are in great demand. In this paper, we consider global sequence features and propose a stacked ensemble learning-based method to predict lncRNAs from transcripts, abbreviated as PredLnc-GFStack. We extract the critical features from the candidate feature list using the genetic algorithm (GA) and then employ the stacked ensemble learning method to construct PredLnc-GFStack model. Computational experimental results show that PredLnc-GFStack outperforms several state-of-the-art methods for lncRNA prediction. Furthermore, PredLnc-GFStack demonstrates an outstanding ability for cross-species ncRNA prediction.

## 1. Introduction

In the last two decades, a massive amount of novel transcript data was discovered due to the development of high-throughput sequencing techniques [1]. The concept of non-coding RNAs (ncRNAs) is generally employed for RNAs that do not encode proteins. Long non-coding RNAs (lncRNAs) refer to ncRNAs with the length exceeding 200 nucleotides [2,3]. Sequencing techniques have shown that there are a lot of lncRNAs in mammals. LncRNAs have been presumed to have no biological functions due to the fact that they could not encode proteins. However, in recent years, in vivo experiments demonstrated that lncRNAs play a vital role in the regulation of gene transcription [4], epigenetic modifications [5], aging [6], cancer [7,8] and many other biological processes [9,10,11]. For example, the transcription of Damage Induced Noncoding (DINO) lncRNA was activated by DNA damage [12,13]. In addition, lncRNAs also guide the enzyme activities and serve as a location transferor [14]. There have been many developed tools associating with lncRNA activity, for example, LADP [15] is a tool for lncRNA-disease association prediction and LPLNP [16], SFPEL-LPI [17] are developed for lncRNA-protein interactions prediction. However, existing in vivo methods for lncRNA classification [18] are often labor-intensive and expensive. Thus, it is indispensable to develop accurate and effective computational methods for the prediction of lncRNAs.

There are a number of computational methods for lncRNA prediction, which can be roughly classified into three categories: the binary classifier-based methods, the deep learning-based methods and the ensemble learning-based methods. The binary classifier-based methods utilize traditional binary classifiers to build prediction models based on two types of transcripts: coding transcripts and non-coding transcripts. The classifier support vector machine (SVM) is most commonly adopted for the lncRNA prediction. Wei et al. [19] proposed an SVM-based method abbreviated as CPC, which assesses the protein-coding potential of a transcript based on multiple sequence features. There are also many SVM-based prediction models, such as CNCI [20], PLEK [21], lncRScan-SVM [22], CPC2 [23], longdist [24] and CPPred [25]. The differences between these methods are the utilization of different features. Random forest (RF) [26] was also adopted for the lncRNA prediction. Achawanantakun et al. [27] proposed LncRNA-ID, which is a method generated by multiple features including characteristics of the putative open reading frames (ORFs), translation scores based on ribosomal coverage, and conservation against characterized protein families for the purpose of lncRNA prediction. COME [28] and FEElnc [29] are also RF-based prediction models. Logistic regression (LR) is also a commonly adopted classifier for the lncRNA prediction, such as Cristiano et al.’s work [30] and CPAT [31]. Deep learning-based methods use deep learning techniques to build prediction models. Fan et al. [32] came up with a method called lncRNA-MFDL, which constructs a deep learning model by fusing ORFs, k-mer, the secondary structure and the most-like coding domain sequences to discriminate lncRNAs and mRNAs. Other deep learning-based methods, such as lncRNAnet [33] and LncADeep [34] have been proposed in the last two years. Ensemble learning-based methods integrate different features, different models or different data to construct prediction models. For example, Hu et al. [35] proposed a two-layer classifier named TLCLnc to distinguish lncRNAs from mRNAs using sequence features: k-mer and sequence-order correlation coefficient factors. There are other ensemble learning-based methods, such as Simopoulos et al.’s work [36] and LncRNApred [37].

Existing methods for lncRNA identification make use of different machine learning methods and/or different features. While all the methods mentioned above are exceedingly effective and innovative, they mostly lack an overall consideration of features for lncRNA prediction, despite that Ventola et al. [38] performed a systematic assessment of a wide collection of features extracted from sequence data. The problem is that different models often employ different features in practice without experimenting on other features of lncRNAs. In addition, most of lncRNA prediction methods just select the features subjectively without using an effective feature selection method to consider various combinations of features, and use single classifier (e.g., SVM, RF) which still has the room for improvement on generalization performance. Aimed at the problems above, we take plenty of features into consideration, adopt a novel feature selection method and use ensemble strategy to construct our model.

In this paper, we consider global sequence features and propose a stacked ensemble learning-based method to differentiate long non-coding RNAs and coding RNAs, abbreviated as PredLnc-GFStack. To start with, we collect sequence-derived features from six categories with thirty-four groups in total, which are all adopted by previous literature regarding lncRNA prediction. We utilize the genetic algorithm to select optimal feature subsets from the candidate feature list, using the area under the curve (AUC) score of the random forest model as the fitness score. Then, we train different random forest models based on multiple optimal subsets, and combine them to build the stacked ensemble model for the lncRNA prediction. PredLnc-GFStack can accept sequences within a length range as inputs, and then predict whether they are long non-coding RNAs or coding RNAs. 

The source code and datasets are available from the following repository at GitHub: https://github.com/BioMedicalBigDataMiningLab/PredLnc-GFStack/.

## 2. Datasets and Method

### 2.1. Datasets

To conduct the study, we attempt to collect two types of sequences: long non-coding RNAs and coding RNAs. The datasets we choose are the human (*Homo sapiens*) data (Release 29, GRCh38.p12) and mouse (*Mus musculus*) data (Release M20, GRCm38.p6) downloaded from GENCODE [39], which are made by merging the manual gene annotation produced by the Ensembl-Havana team and the Ensembl-genebuild automated gene annotation [40]. GENCODE aims to annotate all gene features in human genes by combining manual curation, computational analysis and experimental validation [39]. The download links of datasets can be found in Table S1.

To pre-process the raw data, we first select the transcripts with the length exceeding 200 base pairs (bp) and under 3000 bp from the original datasets. Then we adopt a program called CD-HIT [41,42] to cluster sequences in order to alleviate the problem of redundancy and alignment by removing sequences with ≥80% similarity. After pre-processing, 37260 coding RNAs and 21799 lncRNAs of human are obtained, likewise, 25487 coding RNAs and 13246 lncRNAs of the mouse are obtained. We divide the processed data into two datasets for human and mouse respectively and name them Human-Main, Human-Independent, Mouse-Main, Mouse-Independent. The independent datasets contain 1500 coding RNAs and 1500 lncRNAs randomly selected from the processed datasets, and they are used to select optimal features. The main datasets contain other transcripts from the processed datasets, and they are used for cross-validation.

For cross-species prediction, we respectively train our models on the main datasets for human and mouse, then the models are tested on other publicly available datasets (http://www.rnabinding.com/CPPred/) of multiple species containing human, mouse, zebrafish (*Danio rerio*), fruit fly (*Drosophila melanogaster*), *S. cerevisiae*, nematode (*Caenorhabditis elegans*) and thale cress (*Arabidopsis thaliana*). We name the testing datasets as Human-Testing, Mouse-Testing, Zebrafish-Testing, Fruit-fly-Testing, *S.cerevisiae*-Testing and Integrate-Testing. All datasets are summarized in Table 1.

### 2.2. Features Extraction

In this section, we briefly introduce six types of sequence-derived (sequence intrinsic) features, which can be used for the lncRNA prediction.

Codons are important indicators for transcripts coding into proteins. There are two widely used features about stop codons: stop codon count and stop codon frequency. The stop codon count is the number of stop codons in a transcript and the stop codon frequency means the frequency of stop codons in a transcript. We also calculate the stop codon frame score and stop codon frequency frame score, which mean the variance of stop codon count and frequency among three reading frames. We also consider the Fickett TESTCODE score, which is transformed from the nucleotide position frequencies and bases composition of a transcript by a lookup table. The Fickett TESTCODE score has been proved to enhance the performance of lncRNA prediction [31]. The nucleotide position frequencies [34], which show the contribution of each base among codon positions, are also considered.

There are also many features about ORF of transcripts: the first ORF length, the longest ORF length, ORF coverage, ORF integrity, ORF frame score and the entropy density profiles (EDP) [34] of ORF. The first ORF length and the longest ORF length are the lengths of the first and longest ORF. The ORF coverage is the ratio of longest ORF length and transcript length, and the ratio of first ORF to a transcript length is also included in candidates. The ORF integrity means whether the longest ORF starts with a start codon and ends with a stop codon, and the ORF frame score is the variance of ORF length among ORFs. The EDP of ORF well describes the coding potential of transcripts based on the sequence composition and k-mer.

The guanine-cytosine content (GC content) shows the importance in the prediction of lncRNAs. GC content is the percentage of nitrogenous bases on a DNA or RNA molecule that are either guanine or cytosine. We calculate the GC content of transcripts and GC content in the first, second and third position of codons as GC, GC1, GC2 and GC3. The variance of them among three reading frames can also be considered. We also calculate the GC content of untranslated regions (UTR) [43]. The UTR are two sections which don’t form the protein-coding region in the transcripts, but the features of UTR indicate the coding potential of sequences [34].

The features of coding sequences (CDS) from the transcripts are also considered, including the length of coding sequences, CDS percentage and coding potential of the transcripts. CDS length refers to the length of the coding sequence (CDS) with the most likelihood and CDS percentage is the CDS length divided by the transcript length. The features CDS length, CDS percentage and coding potential of the transcripts are calculated by the program txCdsPredict from UCSC (https://github.com/ENCODE-DCC/kentUtils/blob/master/src/hg/txCds/txCdsPredict/ txCdsPredict.c).

We also consider features about the order of base permutation and composition of transcripts, including transcript length, k-mer, CTD, Hexamer score, Signal to noise ratio (SNR), UTR coverage, transcript length and EDP of transcripts [34]. The k-mer features are the frequencies of different matches of the k adjacent bases. In this study, we set k equals 1,2,3 respectively, and calculate the k-mer features in both sequences and their longest ORF. CTD [44] calculates the frequencies of A, C, G, T in the transcripts, frequencies of the conversion of A, C, G, T between adjacent positions and five relative positions (0%, 25%, 50%, 75%, 100%) of A, C, G, T along the transcripts. Hexamer score [31] is calculated based on in-frame hexamer frequency of coding and noncoding transcripts, and a positive value indicates a protein-coding transcript, whereas a negative value indicates a non-coding transcript. Signal to noise ratio (SNR) [37] utilizes the discrete Fourier Transform on the sequences to distinguish lncRNAs. Owing to the apparent differences shown in SNR between lncRNAs and protein-coding sequences, it is used to distinguish lncRNAs and protein-coding sequences. The UTR coverage means the ratio of 5’ or 3’ UTR length and transcript length. Transcript length and the EDP of the transcript are also considered.

There are several features that reflect the structural properties of transcripts. The Mw means the molecular weight of the predicted peptide and the pI means the theoretical isoelectric point of the predicted peptide. The pI/Mw shows the log 10 transformed ratio of pI and Mw, and it is also applied to the ORFs as pI/Mw frame score. Moreover, Gravy and Instability index, which mean the grand average of hydropathicity and the stability of predicted peptide, are also used.

Therefore, we obtain the features from six categories with 260 dimensions for this study. All features and their dimensions are summarized in Table 2.

### 2.3. Feature Selection by Genetic Algorithm and Random Forest (GA-RF)

In this section, we propose a novel feature selection algorithm that combines the genetic algorithm (GA) [45] and the random forest (RF) model [26], which we name GA-RF. To begin with, we provide a brief introduction of GA and RF. GA is an adaptive method for solving optimization problems by simulating natural evolutionary processes in genetics [45]. It uses the multi-point strategy to search the global optimal solution, so it is less likely to stuck in a locally optimal solution than the algorithms based on the one-point search strategy. RF [26] is one of the most commonly adopted classifiers for lncRNA prediction methods including lncRNA-ID [27], COME [28] and FEElnc [29].

**Figure 1:**
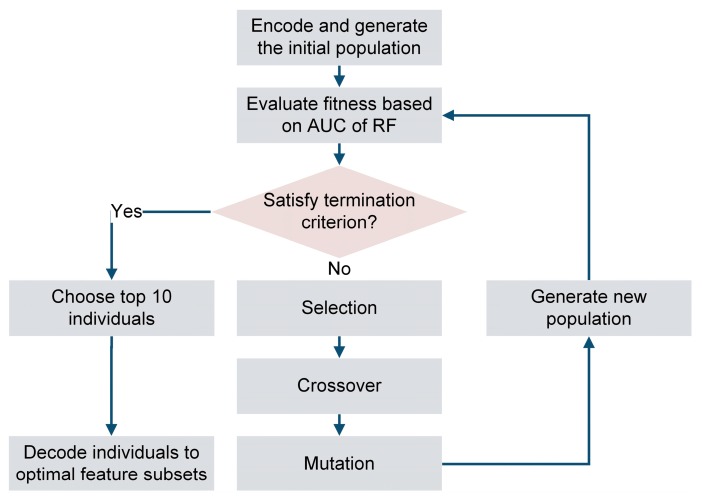
The flowchart of the GA-RF (genetic algorithm-random forest) algorithm.

We encode each candidate feature set using the binary encoding strategy, and randomly take candidate features as feature subsets to generate the initial population. After that, we set the ten-fold cross-validation AUC scores of RF models utilizing different feature subsets as the fitness score of each individual. Moreover, through the update process of selection, crossover and mutation, new individuals are generated to form the next generation. The selection process has an inclination to find the best individual with high fitness score for the next generation. We choose the tournament selection [46] to select the fittest individuals from the current generation and pass them to the next one. The crossover means the “child” individuals would inherit many characteristics of their “parent” individuals, and the “child” solutions are more likely to have higher fitness score. We adopt the single-point crossover and the offspring will be formed with two continuous parts of parents. Mutation ensures the diversity in the population via the displacement of some gene individuals and we use the filpbit strategy, which inverses the bit of the specific genes (https://deap.readthedocs.io/ en/master/api/tools.html#deap.tools.mutFlipBit). The update process would iterate until the maximum fitness score tends to be stable or the iteration of the process reaches the maximum generation. At last, we choose the individuals with top 10 AUC scores and decode them into optimal feature subsets. The flowchart of the GA-RF algorithm, is shown in Figure 1.

### 2.4. Stacked Ensemble Learning in PredLnc-GFStack

In machine learning, the generalization ability is vital to prediction models. The ensemble learning [47] methods are acknowledged to have better generalization performances than any of the constituent model alone [17,48,49,50,51,52,53]. In general, the ensemble learning methods use multiple basic classifiers (e.g., SVM, RF) and combine their prediction results with different strategies, such as averaging and voting. In theory, the ensemble learning methods enlarges the hypothesis space and reduce the risk of over-fitting while training the models, so they usually produce accurate and stable results. Due to the excellent generalization ability, the ensemble learning methods have been widely recognized and applied to lncRNA prediction, one example is TLCLnc [35]. There are two key steps for the construction of ensemble learning methods, the first step is to select and initialize the basic classifiers and then the second step is to combine them with specific strategies [47]. There are different kinds of ensemble learning methods. The bagging method [53] uses the bootstrap strategy which selects samples repeatedly with replacement, then the models fit on the samples respectively and are combined by voting or averaging prediction results. The boosting method [54] converts a set of weak learning classifiers to a strong one by assigning the misclassified data a higher weight and reducing the weight of the correctly classified data. It has been proved by several literature [50,55,56,57,58,59] that all the ensemble learning methods mentioned above have outstanding performance in practice.

**Figure 2:**
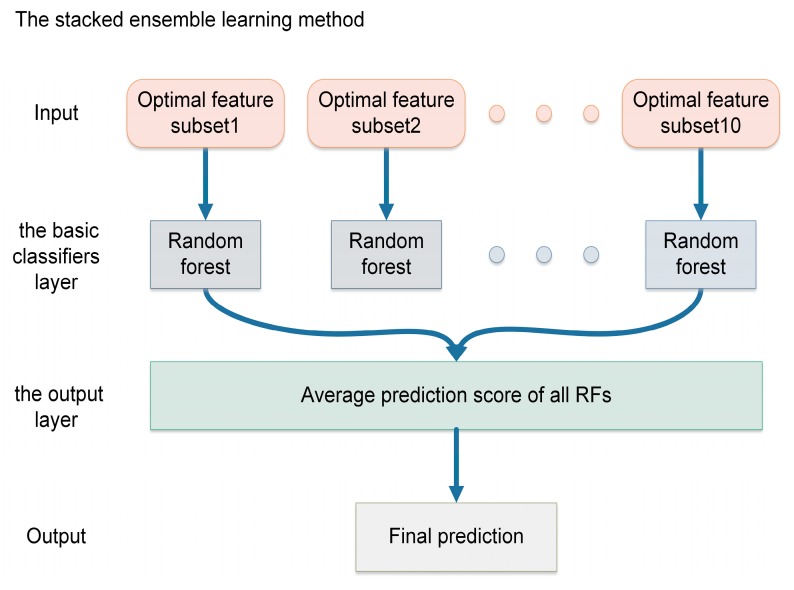
The architecture of the stacked ensemble learning method.

We adopt a stacked ensemble learning method, which has a two-layer structure and is easy to implement. The architecture of our stacked ensemble learning method is shown in Figure 2. We construct a two-layer stacked prediction model containing the basic classifiers layer and the output layer. To be exhaustive, we adopt RF as the basic classifier and then employ the optimal feature subsets to construct 10 basic classifiers, each of them is constructed on a single optimal feature subset. The output layer serves the purpose of calculating the average prediction score of all RFs. In this way, we can effectively avoid the bias of single basic classifier and obtain more balanced performances.

## 3. Results and Discussion

### 3.1. Performance Evaluation

In this section, we employ the 10-fold cross-validation(10-CV) to evaluate all prediction models. A dataset is randomly split into 10 subsets with equal size, and one of these subsets is used as testing dataset while the rest is used as the training dataset for each round of 10-CV. The model is constructed on the training dataset and then makes predictions for the testing dataset. The training and testing processes are repeated until all the subsets are used.

Prediction models are evaluated by several widely recognized evaluation metrics, including the AUC score, the accuracy (ACC), the sensitivity (SN), the specificity (SP), the precision (PRE) and the F1 score (F1), which are defined as follows:
$Accuracy\;\left(ACC\right)=\frac{TP+TN}{TP+TN+FP+FN}$(1)$Sensitivity\;\left(SN\right)=\;\frac{TP}{TP+FN}$(2)$Specificity\;\left(SP\right)=\;\frac{TN}{TN+FP}$(3)$Precision\;\left(PRE\right)=\;\frac{TP}{TP+FP}$(4)$\;\;\;F1\;score\;\left(F1\right)=\;\frac{2\;\times PRE\;\times SN}{PRE+SN}$(5)
where the TP, FN, TN and FP represent the numbers of true positives, false negatives, true negatives and false negatives, respectively. The AUC score is the area under the receiver operating characteristic curve which evaluates the performance regardless of any threshold.

### 3.2. Evaluation of the Optimal Feature Subsets

In this section, we adopt the proposed GA-RF algorithm to select optimal feature subsets on Human-Independent and Mouse-Independent, respectively. GA-RF has two components: GA and RF. For GA, we initialize a population of 500 individuals, and set the number of iterations to 50. For selection, crossover and mutation, we set the tournament selection size to 3, the probability of mating two individuals is set to 0.5, the probability for each gene to be flipped and the probability of mutating an individual are set to 0.05 and 0.2. We set the number of trees to 100 for RF. The fitness score of an individual is the 10-CV AUC score of the RF model using features coded by the individual. We keep the top ten optimal feature subsets respectively for Human-Independent and Mouse-Independent, which will be utilized to construct the stacked ensemble learning model. The AUC scores of models based on optimal feature subsets and sizes of optimal feature subsets are demonstrated in Table 3 for future references.

As shown in Table 3, 10 optimal feature subsets contain different numbers of features, which produce similar AUC scores. The AUC scores of human optimal feature subsets range from 0.94910 to 0.94979, and tend to converge at 0.95, and their sizes range from 127 to 138. The AUC scores of mouse feature subsets range from 0.96322 to 0.96382, and the sizes of mouse feature subsets range from 114 to 127. The average AUC scores of models based on optimal feature subsets for human and mouse are 0.95 and 0.96 respectively. The performances of GA-RF on human are approximately the same as those on mouse despite the slight advantage on the mouse. The average sizes of human optimal feature subsets and mouse optimal feature subsets are 133 and 121, respectively. These results indicate that GA-RF has promising performances for lncRNAs prediction on both human datasets and mouse datasets, i.e., the performance of GA-RF is not affected by the differences of human and mouse with respect to species.

Moreover, we calculate the intersections of human optimal feature subsets and mouse optimal feature subsets to distinguish the critical features selected by the GA-RF. Three kinds of intersections are considered, the intersection of human optimal subsets, the intersection of mouse optimal subsets and the intersection of the combination of the human optimal subsets and mouse optimal subsets. The results are shown in Table S2. The intersection of optimal subsets has a total of 53 features and 37 features for human and mouse, respectively. However, the intersection of optimal subsets for both human and mouse contains only 11 features, including the GC1 frame score, ORF integrity, SNR, pI/Mw, pI, ORF_k_mer_GAT (k-mer on ORF feature), G_pos_fickett (nucleotide position frequency feature), EDP_fea_W (the EDP of transcript feature),EDP_ORF_k_mer_TC (the EDP of ORF feature), CTG (k-mer on transcript feature) and transcript length. Among them, the G_pos_fickett belongs to the codon-related features, ORF integrity and EDP_ORF_k_mer_TC belong to the ORF-related features, GC1 frame score belongs to the GC-related features, the EDP_fea_W, SNR, CTG, ORF_k_mer_GAT and transcript length are part of transcript-related features, the pI and pI/Mw are parts of structure-related features. The features selected by GA-RF come from various categories and are justified to be effective by previous lncRNA prediction models, and thus we can conclude that the optimal feature subsets are predominant and robust. Furthermore, we evaluate the importance of features in the intersection of optimal subsets for both human and mouse (shown in Supplementary Figure S1) using the Sklearn package in Python. PI_Mw has the highest importance score, SNR and G_pos_fickett are followed, they are responsible for the good performance of PredLnc-GFStack than other features.

### 3.3. Evaluation of PredLnc-GFStack on Different Datasets

In this section, the optimal feature subsets for human and mouse generated by GA-RF are adopted to construct the PredLnc-GFStack model respectively. We use the Human-Main and Mouse-Main datasets to construct the models and adopt 10-CV to evaluate the performances of the PredLnc-GFStack models. A comprehensive evaluation of all models should take all the metrics mentioned above into consideration. We list the performances of PredLnc-GFStack on Human-Main and Mouse-Main in Table 4.

As shown in Table 4, PredLnc-GFStack achieves AUC score of 0.956, accuracy of 0.895, sensitivity of 0.884, specificity of 0.901, precision of 0.835 and F1 score of 0.859 on Human-Main and AUC score of 0.969, accuracy of 0.914, sensitivity of 0.875, specificity of 0.933, precision of 0.865 and F1 score of 0.870 on Mouse-Main. From the results, we can observe that these two models not only achieve high accuracy but also achieve the AUC score of 0.956 and 0.969.

Furthermore, we test the performance of PredLnc-GFStack models on multi-species testing datasets. The testing datasets contain ncRNAs from different species are listed in Table 1. We utilize the human optimal feature subsets and mouse optimal feature subsets and adopt the Human-Main and Mouse-Main as training data to construct two PredLnc-GFStack models respectively. Then we use these two PredLnc-GFStack models to predict transcripts in six multi-species datasets. The performances of PredLnc-GFStack models on different species testing datasets are shown in Table 5.

As shown in Table 5, PredLnc-GFStack trained on Human-Main produces AUC scores ranging from 0.971 to 0.995 on multi-species testing datasets, and has the best performance on Human-Testing dataset. PredLnc-GFStack trained on Mouse-Main produces AUC scores ranging from 0.964 to 0.995 on multi-species testing datasets, and has the best performance on Mouse-Testing. These two PredLnc-GFStack models have outstanding performances on the multi-species ncRNA datasets, which indicates that PredLnc-GFStack can effectively predict ncRNAs in a cross-species manner.

What ‘s more, we collected several well-known lncRNAs from some published literature, which focus on the functions of lncRNAs. We tested PredLnc-GFStack on the well-known lncRNA data, and the detailed performances of PredLnc-GFStack can be found in the Table S3. The results showed that PredLnc-GFStack correctly classified all twenty samples, which means PredLnc-GFStack can effectively predict the well-known lncRNAs.

### 3.4. Comparison with Other Methods

**Figure 3:**
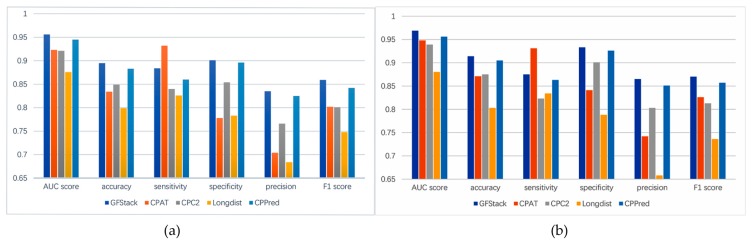
The performance comparison of PredLnc-GFStack and CPAT, CPC2, Longdist, CPPred on Human-Main and Mouse-Main. (**a**) The results of all models evaluated by 10-CV on Human-Main. (**b**)The results of all models evaluated by 10-CV on Mouse-Main.

In this section, we compare PredLnc-GFStack with four benchmark methods, including CPAT [31], CPC2 [23], Longdist [24] and CPPred [25], which are acknowledged for their high accuracy. CPAT utilized an alignment-free logistic regression model and adopted features including ORF size, ORF coverage, Fickett TESTCODE score and hexamer score. CPC2 utilized the SVM model and adopted features including Fickett TESTCODE score, ORF length, ORF integrity and pI. Longdist utilized SVM classifier and adopted features including k-mer selected by PCA and ORF lengths. CPPred utilized CTD features as well as the features of CPAT and CPC2 and constructed an SVM model. We implement these methods using their publicly available source codes, and fairly compare them with our method PredLnc-GFStack. All prediction methods are evaluated by 10-CV on Human-Main and Mouse-Main datasets. The results of all methods are shown in Figure 3.

As shown in Figure 3, PredLnc-GFStack trained on Human-Main and Mouse-Main perform better than CPAT, CPC2, Longdist, CPPred in terms of AUC score. From our perspective, there are quite a few reasons for better performances. First, multiple features are extracted from transcripts, which guarantees the diversity of the model. Second, the optimal feature subsets selected by GA-RF contains a variety of features whose redundancy or noises have been reduced, and it contributes to the enhancement of model performances. At last, the stacked ensemble learning strategy in PredLnc-GFStack guarantees the high accuracy and robustness of the model.

**Figure 4:**
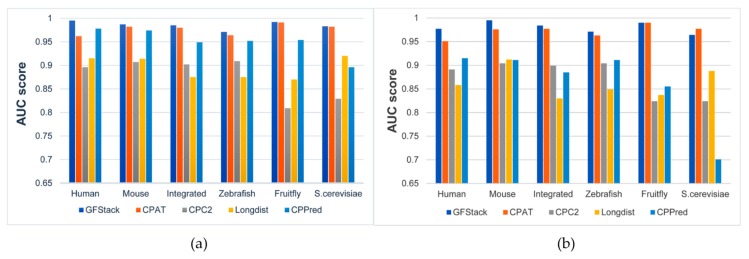
The performances (AUC scores) of all models in cross-species prediction. (**a**) The results of all models trained on Human-Main. (**b**)The results of all models trained on Mouse-Main.

Then we also test PredLnc-GFStack and other four benchmark methods on the ncRNAs multiple-species testing datasets, including Human-Testing, Mouse-Testing, Zebrafish-Testing, Fruit-fly-Testing, *S.cerevisiae*-Testing and Integrate-Testing. The models are trained on Human-Main and Mouse-Main respectively and tested on the multi-species testing datasets. The results of all methods in cross-species prediction are shown in Figure 4.

As shown in Figure 4, PredLnc-GFStack has better AUC scores compared with CPAT, CPC2, Longdist and CPPred on different species testing datasets, despite the fact that the AUC score of PredLnc-GFStack on *S.cerevisiae*-Testing is a little lower than CPAT. In conclusion, PredLnc-GFStack demonstrates better performances on multiple ncRNA datasets than other benchmark methods, and the results reveal PredLnc-GFStack is not only suitable for lncRNA prediction but also for ncRNA prediction.

## 4. Conclusions

In computational biology, the prediction of lncRNAs from transcripts is an important research topic. In this study, we propose a method called PredLnc-GFStack for lncRNA prediction. One of the highlights of PredLnc-GFStack is that it takes global sequence features into consideration. The global sequence features contain a vast variety of feature types, including codon-related features, ORF-related features, GC-related features, coding sequence-related features, transcript-related features and structure-related features, which have already been proved to contribute to the enhancement of prediction performances. Another highlight of PredLnc-GFStack is that it innovatively utilizes an optimal feature selection algorithm GA-RF and a stacked ensemble learning strategy. GA-RF selects the top 10 optimal feature subsets to build the basic classifiers, and a stacked ensemble learning strategy combines these predictors to develop the final lncRNA prediction model. The experiments demonstrate that PredLnc-GFStack not only performs well on lncRNA prediction, but also effectively predicts ncRNAs in a cross-species manner.

In the study, PredLnc-GFStack models are constructed based on the sequences ranging from 200 bp to 3000 bp. Although there exit some lncRNAs shorter than 200 bp or longer than 3000 bp, most known lncRNAs fall in the range. For a given RNA sequence which meets the length range, PredLnc-GFStack models predict its potential of being a lncRNA sequence. In fact, PredLnc-GFStack can be applied to shorter or longer RNA sequences if training datasets contain such sequences.

## Figures and Tables

**Table 1:** Summary of datasets.

Data Sources	Name	Coding RNAs	NcRNAs
GENCODE	Human-Main	35760	20299
	Human-Independent	1500	1500
	Mouse-Main	23987	11746
	Mouse-Independent	1500	1500
preprocess CPPred	Human-Testing	8557	8241
	Mouse-Testing	31102	19930
	Zebrafish-Testing	15594	10662
	Fruit-fly-Testing	17400	4098
	*S.cerevisiae*-Testing	6713	413
	Integrate-Testing	13903	13903

NcRNA: Non-coding RNA.

**Table 2:** All features considered in this paper and their dimensions.

Types	Features (Dimension)
codon-related features	stop codon count (1), stop codon frequency (1), stop codon frame score (1), stop codon frequency frame score (1), nucleotide position frequencies (4), Fickett TESTCODE score (1)
Open reading frame (ORF)-related features	the first ORF length (1), the longest ORF length (1), the ORF coverage (2), the ORF integrity (1), ORF frame score (1), the entropy density profiles (EDP) of ORF (16)
GC-related features	GC (1), GC1 (1), GC2 (1), GC3 (1), GC frame score (1), UTR GC content (2)
coding sequence-related features	Coding sequence (CDS) length (1), CDS percentage (1), coding potential of the transcripts (CDS score) (1)
transcript-related features	transcript length (1), *k*-mer (168), CTD (20), Hexamer score (1), Signal to noise ratio (SNR) (1), untranslated region (UTR) coverage (2), EDP (20)
structure-related features	Molecular weight (Mw) (1), isoelectric point (pI) (1), pI/Mw (1), pI/Mw frame score (1), Gravy (1), Instability index (1)

**Table 3:** Area under the curve (AUC) scores of models based on optimal feature subsets and sizes of optimal feature subsets for human and mouse.

Optimal Feature Subset No.	Human		Mouse	
	AUC	Number of Features	AUC	Number of Features
1	0.94979	134	0.96382	118
2	0.94946	137	0.96350	125
3	0.94940	131	0.96343	127
4	0.94934	136	0.96334	123
5	0.94929	138	0.96327	114
6	0.94929	134	0.96324	123
7	0.94923	129	0.96323	115
8	0.94916	127	0.96323	121
9	0.94913	137	0.96322	122
10	0.94910	128	0.96322	119

**Table 4:** The performances of PredLnc-GFStack on Human-Main and Mouse-Main using 10-CV.

Dataset	AUC	ACC	SN	SP	PRE	F1
Human	0.956	0.895	0.884	0.901	0.835	0.859
Mouse	0.969	0.914	0.875	0.933	0.865	0.870

**Table 5:** The performances of PredLnc-GFStack models on multi-species testing datasets.

Training Dataset	Testing Dataset	AUC	ACC	SN	SP	PRE	F1
Human-Main	Human-Testing	0.995	0.968	0.962	0.974	0.973	0.967
	Mouse-Testing	0.987	0.941	0.879	0.981	0.968	0.921
	Integrated-Testing	0.985	0.907	0.831	0.982	0.979	0.899
	Zebrafish-Testing	0.971	0.901	0.772	0.989	0.980	0.863
	Fruit-fly-Testing	0.992	0.940	0.714	0.993	0.962	0.819
	*S.cerevisiae*-Testing	0.983	0.960	0.828	0.969	0.621	0.710
Mouse-Main	Human-Testing	0.977	0.887	0.807	0.964	0.955	0.875
	Mouse-Testing	0.995	0.944	0.869	0.992	0.985	0.924
	Integrated-Testing	0.984	0.871	0.757	0.985	0.981	0.855
	Zebrafish-Testing	0.971	0.843	0.626	0.991	0.979	0.764
	Fruit-fly-Testing	0.990	0.917	0.593	0.994	0.957	0.733
	*S.cerevisiae*-Testing	0.964	0.942	0.382	0.976	0.500	0.433

## Supplementary Materials

The following are available online at https://www.mdpi.com/2073-4425/10/9/672/s1, Figure S1: The importance scores of features in the intersection of optimal subsets for both human, Table S1: The download links of datasets we used, Table S2: The intersections of optimal feature subsets for human and mouse, Table S3: The performance of PredLnc-GFStack on the list of well-known lncRNAs.
