# Supplementary material for: PredLnc-GFStack: A Global Sequence Feature Based on a Stacked Ensemble Learning Method for Predicting lncRNAs from Transcripts
Source: Genes (Basel). 2019 Sep 3;10(9):672. doi: 10.3390/genes10090672 (PMC6770532; doi:10.3390/genes10090672)
Supplement: Supplementary file 1 [file genes-10-00672-s001.pdf]

Table S1. The download links of datasets we used

Datasets	Download Links of Protein-coding transcript sequences	Download Links of Long non-coding RNA transcript sequences
human (Homo sapiens) data (Release 29, GRCh38.p12)	ftp://ftp.ebi.ac.uk/pub/databases/gencode/Gencode_human/release_29/gencode.v29.pc_transcripts.fa.gz	ftp://ftp.ebi.ac.uk/pub/databases/gencode/Gencode_human/release_29/gencode.v29.lncRNA_transcripts.fa.gz
mouse (Mus musculus) data (Release M20, GRCm38.p6)	ftp://ftp.ebi.ac.uk/pub/databases/gencode/Gencode_mouse/release_M20/gencode.vM20.pc_transcripts.fa.gz	ftp://ftp.ebi.ac.uk/pub/databases/gencode/Gencode_mouse/release_M20/gencode.vM20.lncRNA_transcripts.fa.gz

Table S2. The intersections of optimal feature subsets for human and mouse

Species	Intersections of optimal feature subsets	Descriptions	Types
Human	A_pos_fickett, G_pos_fickett,	nucleotide position frequencies	codon-related features
	fickett_score	Fickett TESTCODE score	
	longest_orf_Rlen	the longest ORF coverage	ORF-related features
	integrity	whether the longest ORF starts with a start codon and ends with a stop codon	
	EDP_ORF_k_mer_GA, EDP_ORF_k_mer_CG, EDP_ORF_k_mer_CT, EDP_ORF_k_mer_TC	The EDP (entropy density profiles) of ORF	
	transcript_GC	GC content of transcript	GC-related features
	GC1_frame_score, GC3_frame_score	The variance of GC content among three reading frames	
	C1, G1, G2	CTD features	transcript-related features
	transcript_length	The length of transcript	
	hexamer_score	Features calculated based on in-frame hexamer frequency	
	snr	Signal to noise ratio (SNR)	
	EDP_fea_W, EDP_fea_Y	The EDP (entropy density profiles) of transcript	
	ACC, C, GCC, GCT, CTG, AAT, GAA, TT, GGA, GTC, CTC, ORF_k_mer_AGT,	k-mer (the frequencies of different matches of	

	ORF_k_mer_CTA, ORF_k_mer_GA, ORF_k_mer_CGA, ORF_k_mer_GAT, ORF_k_mer_TCG, ORF_k_mer_AAT, ORF_k_mer_TAT, ORF_k_mer_AA, ORF_k_mer_CAC, ORF_k_mer_CGT, ORF_k_mer_AAC, ORF_k_mer_TAC, ORF_k_mer_CC, ORF_k_mer_GGG, ORF_k_mer_GTC, ORF_k_mer_ATC, ORF_k_mer_TAA	the k adjacent bases) on transcript and ORF	
	pI	The theoretical isoelectric point of the predicted peptide	structure-related features
	PI/Mw	The log 10 transformed ratio of pI and Mw (molecular weight)	
	pI/Mw_frame_score	pI/Mw value of ORF	
	Gravy	the grand average of hydropathicity	
	G_pos_fickett	nucleotide position frequencies	codon-related features
	STOP_Codon_Count	the number of stop codons in a transcript	
	integrity	whether the longest ORF starts with a start codon and ends with a stop codon	ORF-related features
	EDP_ORF_k_mer_AG, EDP_ORF_k_mer_TC	The EDP (entropy density profiles) of ORF	
Mouse	ORF_frame_score	the variance of ORF length among ORFs	
	GC1_frame_score	The variance of GC content among three reading frames	GC-related features
	UTR5_GC_content	the GC content of untranslated regions (UTR)	
	txCdsPredict_score	coding potential of the transcripts are calculated by the program txCdsPredict	coding sequence-related features
	transcript_length	The length of transcript	

	TAT, TGT, TAG, CTG, ATG, TA, GC, AC, CGC, GAT, TG, TTG, AA, TAA, ORF_k_mer_GTG, ORF_k_mer_CG, ORF_k_mer_GAT, ORF_k_mer_CT, ORF_k_mer_AGA, ORF_k_mer_CCA,	k-mer (the frequencies of different matches of the k adjacent bases) on transcript and ORF	transcript-related features
	snr	Signal to noise ratio (SNR)	
	EDP_fea_A, EDP_fea_W, EDP_fea_L, EDP_fea_C	The EDP (entropy density profiles) of transcript	
	pI	The theoretical isoelectric point of the predicted peptide	structure-related features
	PI/Mw	The log 10 transformed ratio of pI and Mw (molecular weight)	
	G_pos_fickett	nucleotide position frequencies	codon-related features
	integrity	whether the longest ORF starts with a start codon and ends with a stop codon	ORF-related features
	EDP_ORF_k_mer_TC	The EDP (entropy density profiles) of ORF	
	GC1_frame_score	The variance of GC content among three reading frames	GC-related features
Human and Mouse	transcript_length	The length of transcript	transcript-related features
	snr	Signal to noise ratio (SNR)	
	CTG, ORF_k_mer_GAT	k-mer (the frequencies of different matches of the k adjacent bases) on transcript and ORF	
	EDP_fea_W	The EDP (entropy density profiles) of transcript	
	pI	The theoretical isoelectric point of the predicted peptide	structure-related features

	PI/Mw	The log 10 transformed ratio of pI and Mw (molecular weight)

Table S3. The performance of PredLnc-GFStack on the list of well-known lncRNAs

LncRNA ID	LncRNA Name	References	Prediction results
NR_130791.1	lncRNA-JADE	[1]	lncRNA
NR_144384.1	DINOL	[2]	lncRNA
NR_036469.2	Linc p21	[3]	lncRNA
KT318134.1	DDSR1	[4]	lncRNA
NR_027451.1	NORAD	[5]	lncRNA
NR_038366.1	HOTAIRM1	[6]	lncRNA
NR_024582.1	Xist	[7]	lncRNA
JF519002.1	Tsix	[8]	lncRNA
KX036209.1	NEAT1	[9]	lncRNA
NR_028261.1	Rian	[10]	lncRNA
NR_047517.1	HOTAIR	[11]	lncRNA
NR_045680.1	HEIH	[12]	lncRNA
NR_036484.1	FEZF1-AS1	[13]	lncRNA
NR_046466.1	MEG3	[14]	lncRNA
NR_015379.3	UCA1	[15]	lncRNA
NR_110375.1	THRIL	[16]	lncRNA

NR_045420.2	Bvht	[17]	lncRNA
NR_045471.2	Fendrr	[18]	lncRNA
NR_148381.1	PNKY	[19]	lncRNA
NR_131221.1	SPRY4-IT1	[20]	lncRNA

Figure S1. The importance scores of features in the intersection of optimal subsets for both human. The x-axis means the features in the intersection of optimal subsets for both human and mouse, and y-axis means the importance scores of corresponding features, which is calculated using Sklearn package in Python.

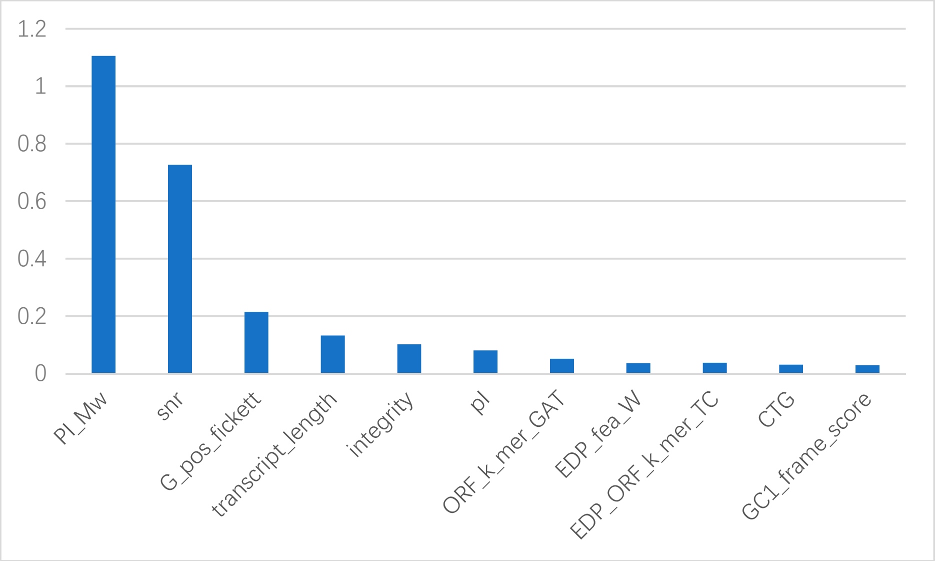

Reference

1. Wan, G.; Hu, X.; Liu, Y.; Han, C.; Sood, A.K.; Calin, G.A.; Zhang, X.; Lu, X. A novel non-coding RNA lncRNA-JADE connects DNA damage signalling to histone H4 acetylation. *EMBO J* **2013**, *32*, 2833–2847.
2. Schmitt, A.M.; Garcia, J.T.; Hung, T.; Flynn, R.A.; Shen, Y.; Qu, K.; Payumo, A.Y.; Peres-da-Silva, A.; Broz, D.K.; Baum, R. An inducible long noncoding RNA amplifies DNA damage signaling. *Nat Gen* **2016**, *48*, 1370.
3. Xia, W.; Zhuang, L.; Hou, M. Role of lincRNA-p21 in the protective effect of macrophage inhibition factor against hypoxia/serum deprivation-induced apoptosis in mesenchymal stem cells. *Int J Mol Med* **2018**, *42*, 2175–2184.
4. Sharma, V.; Khurana, S.; Kubben, N.; Abdelmohsen, K.; Oberdoerffer, P.; Gorospe, M.; Misteli, T. A *BRCA1*-interacting lncRNA regulates homologous recombination. *EMBO Rep* **2015**, *16*, 1520–1534.
5. Miao, Z.; Guo, X.; Tian, L. The long noncoding RNA NORAD promotes the growth of gastric cancer cells by sponging miR-608. *Gene* **2019**, *687*, 116–124.
6. Xiao, Y.; Yan, X.; Yang, Y.; Ma, X. Downregulation of long noncoding RNA HOTAIRM1 variant 1 contributes to osteoarthritis via regulating miR-125b/BMPR2 axis and activating JNK/MAPK/ERK

pathway. *Biomed Pharmacother* **2019**, *109*, 1569–1577.
7. Lin, X.-q.; Huang, Z.-m.; Chen, X.; Wu, F.; Wu, W. XIST Induced by JPX suppresses hepatocellular carcinoma by sponging miR-155-5p. *Yonsei Med J* **2018**, *59*, 816–826.
8. Shevchenko, A.I.; Malakhova, A.A.; Elisaphenko, E.A.; Mazurok, N.A.; Nesterova, T.B.; Brockdorff, N.; Zakian, S.M. Variability of sequence surrounding the *Xist* gene in rodents suggests taxon-specific regulation of X chromosome inactivation. *PLoS One* **2011**, *6*, e22771.
9. Cornelis, G.; Souquere, S.; Vernochet, C.; Heidmann, T.; Pierron, G. Functional conservation of the lncRNA NEAT1 in the ancestrally diverged marsupial lineage: Evidence for NEAT1 expression and associated paraspeckle assembly during late gestation in the opossum *Monodelphis domestica*. *RNA biology* **2016**, *13*, 826–836.
10. Saito, T.; Hara, S.; Kato, T.; Tamano, M.; Muramatsu, A.; Asahara, H.; Takada, S. A tandem repeat array in IG-DMR is essential for imprinting of paternal allele at the Dlk1–Dio3 domain during embryonic development. *Human molecular genetics* **2018**, *27*, 3283–3292.
11. Chu, Y.-H.; Hardin, H.; Eickhoff, J.; Lloyd, R.V. In situ hybridization analysis of long non-coding RNAs MALAT1 and HOTAIR in gastroenteropancreatic neuroendocrine neoplasms. *Endocrine pathology* **2019**, *30*, 56–63.
12. Cui, C.; Zhai, D.; Cai, L.; Duan, Q.; Xie, L.; Yu, J. Long noncoding RNA HEIH promotes colorectal cancer tumorigenesis via counteracting miR-939–mediated transcriptional repression of Bcl-xL. *Cancer Res Treat* **2018**, *50*, 992.
13. Zhang, Y.; Yang, Q.-X.; Peng, T.-T.; Wang, L.-J.; Xiao, G.-L.; Tang, S.-B. Prognostic value of lncRNA FEZF1 antisense RNA 1 over-expression in oncologic outcomes of patients with solid tumors. *Medicine* **2019**, *98*.
14. Yan, L.; Liu, Z.; Yin, H.; Guo, Z.; Luo, Q. Silencing of MEG3 inhibited ox-LDL-induced inflammation and apoptosis in macrophages via modulation of the MEG3/miR-204/CDKN2A regulatory axis. *Cell Biol Int* **2019**, *43*, 409–420.
15. Guo, N.; Sun, Q.; Fu, D.; Zhang, Y. Long non-coding RNA UCA1 promoted the growth of adrenocortical cancer cells via modulating the miR-298-CDK6 axis. *Gene* **2019**, *703*, 26–34.
16. Wang, Y.; Liu, Y.; Li, Z.; Yan, X.; Huang, C.; Ye, X.; Sun, X.; Qin, S.; Zhong, X.; Zeng, C. Association between MALAT1 and THRIL polymorphisms and precancerous cervical lesions. *Genet Test Mol Biomarkers.* **2018**, *22*, 509–517.
17. Hou, J.; Long, H.; Zhou, C.; Zheng, S.; Wu, H.; Guo, T.; Wu, Q.; Zhong, T.; Wang, T. Long noncoding RNA Braveheart promotes cardiogenic differentiation of mesenchymal stem cells in vitro. *Stem Cell Res Ther* **2017**, *8*, 4.
18. Dong, B.; Zhou, B.; Sun, Z.; Huang, S.; Han, L.; Nie, H.; Chen, G.; Liu, S.; Zhang, Y.; Bao, N. LncRNA-FENDRR mediates VEGFA to promote the apoptosis of brain microvascular endothelial cells via regulating miR-126 in mice with hypertensive intracerebral hemorrhage. *Microcirculation* **2018**, *25*, e12499.
19. Ramos, A.D.; Andersen, R.E.; Liu, S.J.; Nowakowski, T.J.; Hong, S.J.; Gertz, C.C.; Salinas, R.D.; Zarabi, H.; Kriegstein, A.R.; Lim, D.A. The long noncoding RNA Pnky regulates neuronal differentiation of embryonic and postnatal neural stem cells. *Cell stem cell* **2015**, *16*, 439–447.
20. Wu, H.; Wang, Y.; Chen, T.; Li, Y.; Wang, H.; Zhang, L.; Chen, S.; Wang, W.; Yang, Q.; Chen, C. The N-terminal polypeptide derived from vMIP-II exerts its anti-tumor activity in human breast cancer by regulating lncRNA SPRY4-IT1. *Biosci Rep* **2018**, *38*, BSR20180411.

© 2019 by the authors. Submitted for possible open access publication under the terms and conditions of the Creative Commons Attribution (CC BY) license (http://creativecommons.org/licenses/by/4.0/).
